# Nogo-B receptor increases the resistance to tamoxifen in estrogen receptor-positive breast cancer cells

**DOI:** 10.1186/s13058-018-1028-5

**Published:** 2018-09-12

**Authors:** Pin Gao, Xiang Wang, Ying Jin, Wenquan Hu, Yajun Duan, Aiping Shi, Ye Du, Dong Song, Ming Yang, Sijie Li, Bing Han, Gang Zhao, Hongquan Zhang, Zhimin Fan, Qing Robert Miao

**Affiliations:** 1 Department of Breast Surgery, The First Hospital of Jilin University, 71 Xinmin street, Changchun, 130021 Jilin Province China; 2 Division of Pediatric Surgery, Department of Surgery, Children’s Research Institute, Medical College of Wisconsin, 8701 W Watertown Plank Rd, Milwaukee, WI 53226 USA; 3 Division of Pediatric Pathology, Department of Pathology, Children’s Research Institute, Medical College of Wisconsin, 8701 W Watertown Plank Rd, Milwaukee, WI 53226 USA; 4 Department of Human Anatomy, Histology, and Embryology, Key Laboratory of Carcinogenesis and Translational Research (Ministry of Education) and State Key Laboratory of Natural and Biomimetic Drugs, Peking University Health Science Center, Beijing, 100191 China; 5 College of Life Sciences, Nankai University, 94 Weijin Road, Tianjin, 300071 China

**Keywords:** Nogo-B receptor, Survivin, Tamoxifen, Estrogen receptor, Breast cancer

## Abstract

**Backgrounds:**

Tamoxifen is typically used to treat patients with estrogen receptor alpha (ERα)-positive breast cancer. However, 30% of these patients gain acquired resistance to tamoxifen during or after tamoxifen treatment. As a Ras modulator, Nogo-B receptor (NgBR) is required for tumorigenesis through the signaling crosstalk with epidermal growth factor (EGF) receptor (EGFR)-mediated pathways. NgBR is highly expressed in many types of cancer cells and regulates the sensitivity of hepatocellular carcinoma to chemotherapy. In this study, we found the expression of NgBR is increased in tamoxifen-resistant ERα-positive breast cancer cells.

**Methods:**

Tamoxifen-resistant ERα-positive MCF-7 and T47D breast cancer cell lines were established by culturing with gradually increased concentration of 4-hydroxytamoxifen (4-OHT). The effects of NgBR on tamoxifen resistance was determined by depleting NgBR in these cell lines using previously validated small interfering RNA (siRNA). The effects of 4-OHT on cell viability and apoptosis were determined using well-accepted methods such as clonogenic survival assay and Annexin V/propidium iodide staining. The alteration of EGF-stimulated signaling and gene expression was determined by western blot analysis and real-time PCR, respectively.

**Results:**

NgBR knockdown with siRNA attenuates EGF-induced phosphorylation of ERα and restores the sensitivity to tamoxifen in ERα-positive breast cancer cells. Mechanistically, our data demonstrated that NgBR knockdown increases the protein levels of p53 and decreases survivin, which is an apoptosis inhibitor.

**Conclusions:**

These results suggested that NgBR is a potential therapeutic target for increasing the sensitivity of ERα-positive breast cancer to tamoxifen.

**Electronic supplementary material:**

The online version of this article (10.1186/s13058-018-1028-5) contains supplementary material, which is available to authorized users.

## Background

Breast cancer is the most common cancer in women around the world [1, 2]. About 75% of the cases are estrogen receptor alpha (ERα)-positive breast cancer [3]. These patients undergo adjuvant endocrine therapy to increase disease-free survival (DFS) and overall survival (OS) [4]. According to the National Comprehensive Cancer Network (NCCN) guideline, patients with invasive breast cancer who are ERα-positive or progesterone receptor (PR)-positive are eligible for tamoxifen, the selective estrogen receptor modulator (SERM) [5]. Tamoxifen, or its active metabolite 4-hydroxytamoxifen (4-OHT), are reported to induce breast cancer cell apoptosis [6]. However, recurrence within 15 years occurs in one third of patients treated with tamoxifen for 5 years [4].

The underlying mechanisms of developing resistance, especially acquired resistance, to tamoxifen are complex and numerous, including ligand-independent activation of ERα or its co-activators through phosphorylation, and the inhibition of apoptosis through constitutive activation of survival signaling [7]. Clinical evidence shows that patients with human epidermal growth factor receptor 2 (HER2) overexpression and lower ERα levels are more likely to become tamoxifen-resistant [8]. Preclinical studies implicate the contribution of growth factor receptor signaling pathways, such as EGFR and HER2, to tamoxifen resistance [9, 10].

Nogo isoforms, including Nogo-A, Nogo-B and Nogo-C, are members of a reticulon protein superfamily. Nogo-B is mainly expressed in peripheral tissues [11]. NgBR was identified as a Nogo-B receptor specific for the amino terminus of Nogo-B (AmNogo-B). NgBR is necessary for angiogenesis by mediating chemotaxis of endothelial cells [12], and is essential for vasculature development [13, 14]. Our recent findings demonstrated that NgBR binds farnesylated Ras and recruits Ras to the plasma membrane, which is a critical step required for receptor tyrosine kinase (RTK)-mediated activation of Ras signaling in human breast cancer cells and tumorigenesis [15]. Greater expression of NgBR in ERα-positive breast tumor tissues is significantly correlated with expression of survivin [16], which is an apoptosis inhibitor [17]. A proteomic study also showed that NgBR is essential for promoting epithelial-mesenchymal transition (EMT) in breast cancer cells [18]. However, the involvement of NgBR in tamoxifen resistance of ERα-positive breast cancer is still unknown. In this study, we showed that NgBR knockdown attenuates tamoxifen resistance in MCF-7 and T47D breast cancer cells by inhibiting EGF-stimulated phosphorylation of ERα. Also, NgBR knockdown restored the sensitivity of ERα-positive breast cancer cells to tamoxifen through decreasing p53-mediated expression of survivin. Our results suggest that NgBR is a potential therapeutic target for increasing the efficacy of tamoxifen and overcoming the resistance to tamoxifen in ERα-positive patients with breast cancer.

## Methods

### Antibodies, reagents and plasmids

A peptide (AHHRMRWRADGRSLEK, residues from 81 to 96 of NgBR) was used to immunize rabbits (Epitomics, Burlingame, CA, USA). Antiserum was purified using the same peptide-conjugated SulfoLink Coupling Gel (Pierce, Rockford, IL, USA). Purified NgBR rabbit polyclonal antibody was used for immunostaining. The peptide recognizing epitope 14–30 of human Nogo-B was used to immunize rabbits (IMG-5346A, Imgenex, San Diego, CA, USA). Antibodies for NgBR (#ab168351) and phosphorylated ERα (phos-S118) (#ab32396) were purchased from Abcam (Cambridge, MA, USA). Antibodies for phos-EGFR (#3777), phos-Akt (#S473), phos-p42/44 extracellular signal-related kinase (ERK) (#9101), total Akt (#4691), total ERK (#4595), ERα (#8644), HER2 (#4290) and survivin (#2808) were purchased from Cell Signaling Technology (Beverly, MA, USA). We also used antibodies for p53 (#10442–1-AP), β-actin (#60008–1) and Hsp90 (13171–1-AP) from Proteintech (Rosement, IL, USA). EGF (#E5036) was purchased from Sigma-Aldrich (St. Louis, MO, USA). HER2 plasmid DNA was a gift from Mien-Chie Hung (Addgene plasmid # 16257).

### Cell culture

Human breast cancer cell lines MCF-7 and T-47D were obtained from the American Type Culture Collection. The cells were grown in DMEM with L-Glutamine (MCF-7 Gibco) or RPMI-1640 with L-Glutamine (T47D, Gibco) supplemented with 10% FBS (Sigma, St. Louis, MO, USA) and 1% penicillin streptomycin glutamine (Gibco). The tamoxifen-resistant cell lines (MCF-7-TamR and T47D-TamR) were derived from the parental cell lines by continuous gradual exposure to 4-OHT (Sigma, St. Louis, MO, USA) to reach the final concentration of 1 μM in methanol in 6 months [19]. Culture medium was replaced every 2–3 days. Cells were incubated at 37 °C in a humidified atmosphere containing 5% CO2. The cell lots used in this study were authenticated prior to in vitro experiments.

### Small interfering RNA (siRNA) and plasmid transfection

NgBR siRNA (forward, GGAAAUACAUAGACCUACA; reverse, UGUAGGUCUAUGUAUUUCC) oligonucleotides with 3′ dTdT overhangs were synthesized by QIAGEN (Valencia, CA, USA). The specificity of NgBR siRNA has been validated in our previous publication [12]. Control siRNA in experiments refers to a non-silencing (NS) siRNA (NSF, UUCUCCGAACGUGUCACGU; NSR, ACGUGACACGUUCGG AGAA) designed and synthesized by QIAGEN. P53 siRNA (sc44218) and survivin siRNA (sc-29,499) were purchased from Santa Cruz (Dallas, TX, USA). Cells were transfected with siRNA using Lipofectamine RNAiMAX reagent (ThermoFisher Scientific). For transient NgBR overexpression experiments, MCF-7 cells were transfected with pIRES-NgBR-HA plasmid DNA using Lipofectamine 2000 reagent (ThermoFisher Scientific).

### Quantitative real-time polymerase chain reaction

Total RNA was extracted from cells by using TRIzol reagent according to the manual (ThermoFisher Scientific) and complementary DNA (cDNA) was reverse-transcribed using the iScript cDNA Synthesis Kit (Bio-Rad, Hercules, CA, USA) according to the manufacturer’s instructions. Real-time PCR was performed using iTaq Universal SYBR Green Supermix (Bio-Rad, USA) and was run on MyiQ Single Color Real-Time PCR Detection System (Bio-Rad). The relative messenger RNA (mRNA) expression of each gene was normalized to glyceraldehyde-3-phosphate dehydrogenase (GAPDH) RNA levels. The primers were synthesized by Integrated DNA Technologies (Coralville, IA, USA). The forward and reverse primers for NgBR are 5′-tgccagttagtagcccagaagcaa-3′ and 5′-tgatgtgccagggaagaaagccta-3′, respectively. The forward and reverse primers for p53 are 5′-cctttctatcagccccagaggata-3′ and 5′-GGGACATCCTTAATTATCTGGGGT-3′, respectively. The forward and reverse primers for GAPDH are 5′-aacctgccaagtatgatgac-3′ and 5′-tctcttgctcagtgtccttg-3′, respectively. The forward and reverse primers for EGFR are 5′-aagccatatgacggaatccc-3′ and 5′-ggaactttgggcgactatctg-3′, respectively. The forward and reverse primers for ERα are 5′-cgactatatgtgtccagccac-3′ and 5′-cctcttcggtcttttcgtatcc-3′, respectively. The forward and reverse primers for survivin are 5′-caaggagctggaaggctg-3′ and 5′-ttcttggctctttctctgtcc-3′, respectively.

### Clonogenic survival assay

Cells were seeded in triplicate into cell culture dishes (1000 cells/well). MCF-7-TamR or T47D-TamR cells were transfected with NgBR siRNA or non-silencing siRNA. At 12 h after transfection, cells were treated with or without 4-OHT. After 14 days, cells were washed with PBS, fixed with methanol for 15 min and stained with 0.1% crystal violet for 15 min. Colonies containing 50 or more cells were counted [20].

### Cell viability assay

Cell viability was determined using the CCK-8 assay. Cells were seeded at the density of 5000 cells/well into 96-well plates; 10 μL of CCK-8 (Sigma) was added to each well. Then cells were incubated at 37 °C for 1–3 h. The absorbance of the reaction was measured using a plate reader (Molecular Devices, Sunnyvale, CA, USA). Another cell viability assay used in this study was trypan blue staining. Cells were seeded into 96-well plates (5000/well). Then, the cells were incubated for 24 h before transfection with NgBR siRNA. The treatment time with 4-OHT was 3 days. Before staining, cells were washed with PBS and dislodged with trypsin. Then cell suspension and trypan blue were mixed (1:1) for 3 min. The number of viable and dead cells on the hemocytometer were counted under a microscope.

### Apoptosis assay by FITC Annexin V/propidium iodide (PI) staining

An FITC Annexin V Apoptosis Detection kit (# 556547) was purchased from BD Biosciences (San Jose, CA, USA). Cells cultured in 6-well plates were transfected with siRNA of NgBR, p53 or survivin, and then treated with 4-OHT for 48 h. Cells were then stained with 5 μL Annexin V-FITC and 5 μL PI in 500 uL of apoptosis reaction solution at room temperature in the dark for 15 min following the manufacture’s instruction. The BD LSR II flow cytometer was used to detect apoptotic cells.

### Western blot

Cells were harvested and washed with PBS, and then lysed with lysis buffer supplemented with Pierce Protease and Phosphatase Inhibitor Mini Tablets (ThermoFisher Scientific) for 10 min on ice. The whole cell lysate was scraped from the plates and then centrifuged at 12000 rpm for 10 min at 4 °C. The concentrations of protein were determined by BCA Protein Assay Kit (Bio-Rad). Cell lysates were subjected to SDS-PAGE, transferred to nitrocellulose blotting membrane (GE Healthcare Life Sciences, Pittsburgh, PA, USA), then incubated with primary specific antibodies at 4 °C overnight. The membranes were incubated with secondary antibodies (Jackson ImmunoResearch, West Grove, PA, USA) at 1:10000 dilution for 2 h at room temperature. The protein band intensities were evaluated using Amersham ECL Western Blotting Detection System (GE Healthcare Life Sciences) and were normalized to housekeeping genes, either β-actin or HSP90. All western blot experiments were performed at least three times.

### Raf-pull-down assay

Ras activity was assessed using GST-Raf-1 RBD beads (RF02, Cytoskeleton, Denver, CO, USA) according to the manufacturer’s protocol: 500μg total cell lysate was incubated with 10 μL GST-Raf1-RBD beads overnight at 4 °C with gentle rocking. Samples were washed five times, then dissolved in 20 μL 2X SDS sample buffer. Activated H-Ras and K-Ras was determined by western blot using specific H-Ras (GTX116041, GeneTex, San Antonio, TX, USA) or K-Ras (12063–1-AP, Proteintech, Rosemont, IL, USA) antibodies, respectively.

### Tissue microarray slides

Breast cancer tissue was collected from 22 patients at the First Hospital of Jilin University (Changchun, China).

We had consent from all patients for participating in this study. All of the patients received modified radical mastectomy and were diagnosed with infiltrating ductal carcinoma via pathological diagnosis. Immunohistochemistry was performed to examine NgBR, Nogo-B, and survivin expression levels following standard methodology described in our previous publication [16]. All of these breast cancer cases were histopathologically re-evaluated on hematoxylin and eosin-stained slides by two pathologists. The breast tissue specimens are anonymous. The study was approved by the ethical committee of the First Hospital of Jilin University.

### Association between NgBR or survivin expression and survival in patients with breast cancer 

All data were collected from a public online clinical database (http://kmplot.com). We analyzed the association between mRNA level of NgBR (NUS1, 225071_x at from Kaplan–Meier Plot database) or survivin (BIRC5, 202094_x at from Kaplan–Meier Plot database) and survival in patients with breast cancer. Kaplan-Meier survival curves according to NgBR expression status were used to analyze the relapse-free survival (RFS) and log-rank *p* values (SPSS 23.0 USA).

### Statistical analysis

Data were analyzed from at least three independent experiments. The results were reported as the mean ± SD. Values of *p* < 0.05 were considered statistically significant. Student’s *t* test or analysis of variance (ANOVA) were performed as appropriate. Correlation between NgBR and survivin expression was analyzed using Fisher’s test. Statistical analyses were performed using Prism 6.0 software (GraphPad software, USA).

## Results

### NgBR expression is increased in tamoxifen-resistant breast cancer cells

**Fig. 1:**
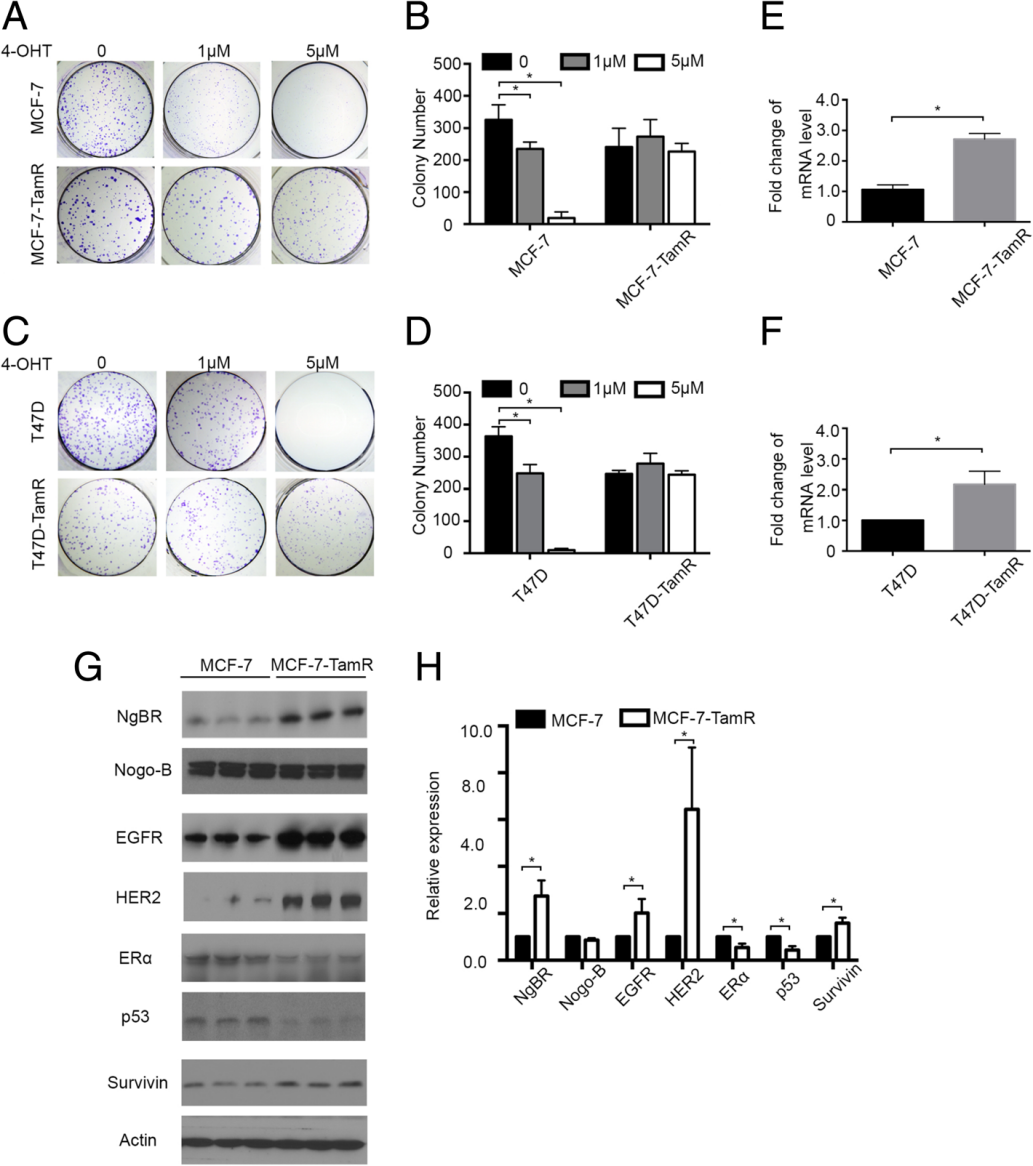
Nogo-B receptor (NgBR) is highly expressed in the tamoxifen resistant MCF-7-TamR and T47D-TamR cells. **a** Colony formation assay was performed as described in “Methods”. Wild-type MCF-7 and tamoxifen-resistant MCF-7-TamR cells were treated with different concentrations of 4-OHT (0, 1 and 5 μM). **b** Quantification of colony number presented in colony formation assays of MCF-7 and MCF-7-TamR cells. **c** Colony formation assay of wild-type T47D and tamoxifen-resistant T47D-TamR cells treated with different concentrations of 4-OHT (0, 1 and 5 μM). **d** Quantification of colony number in colony formation assays of T47D and T47D-TamR cells. **e**, **f** mRNA level of NgBR was increased in MCF-7-TamR and T47D-TamR cells as compared to wild-type MCF-7 and T47D cells, respectively. The relative amount of NgBR mRNA level was normalized to glyceraldehyde-3-phosphate dehydrogenase (GAPDH). **g** NgBR protein level was increased in MCF-7-TamR cells. Protein levels of Nogo-B, epidermal growth factor receptor (EGFR), human epidermal growth factor receptor 2 (HER2), estrogen receptor alpha (ERα), p53 and survivin in MCF-7 and MCF-7-TamR cells were determined using western blot analysis. **h** Quantitative analysis of protein levels using ImageJ and normalized to the housekeeping gene β-actin. Data are presented as fold changes in MCF-7-TamR cells compared to MCF-7 cells. The data are from three separate repeat experiments, and are presented as the mean ± SD (*p < 0.05, *n* = 3)

Tamoxifen resistant MCF-7 (MCF-7-TamR) and T47D (T47D-TamR) ERα-positive breast cancer cells were established following the previously described method [19]. To validate tamoxifen resistance in established MCF-7-TamR and T47D-TamR cells, both normal and tamoxifen-resistant cells were treated with 0–5 μM 4-OHT. As shown in Fig. 1a–d, 5 μM 4-OHT cannot attenuate the colony formation capability of MCF-7-TamR and T47D-TamR cells. However, parental cells cannot survive treatment with 5 μM 4-OHT. CCK-8 cell viability assay was also used for determining the response of these breast cancer cells to tamoxifen (Additional file 1: Figure S1A and B). Similarly, both MCF-7-TamR and T47D-TamR can survive treatment with 5 μM 4-OHT. The levels of NgBR transcript and protein were determined by real-time PCR (Fig. 1e and f) and western blot analysis (Fig. 1g and h). The expression of NgBR was increased in both MCF-7-TamR (Fig. 1e, g and h) and T47D-TamR cells (Fig. 1f; Additional file 2: Figure S2) as compared to that in their parental cells. The alteration of other gene expression between MCF-7 and MCF-7-TamR cells is shown in Fig. 1g and h. Consistent with many previous studies [19, 21, 22], we also noted increased expression of EGFR, HER2, and survivin, and decreased expression of p53 and ERα in MCF-7-TamR (Fig. 1g and h).

### NgBR knockdown attenuates the tamoxifen resistance

**Fig. 2:**
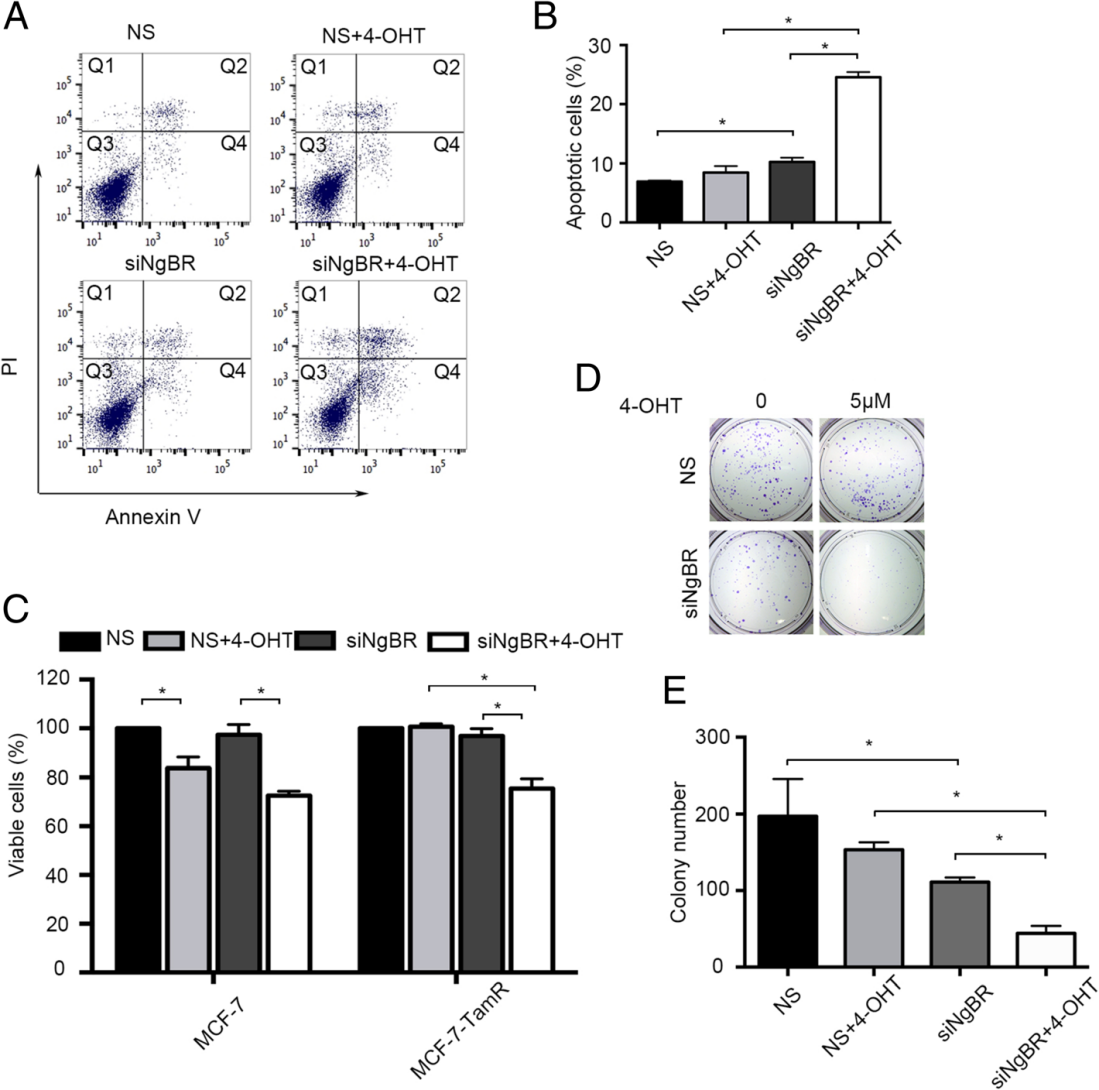
Nogo-B receptor (NgBR) knockdown decreases the resistance of MCF-7-TamR cells to tamoxifen. **a** NgBR knockdown increases 4-OHT-induced apoptosis of MCF-7-TamR cells; 4-OHT, 5 μM. The apoptotic cells were detected by Annexin V-PI staining as described in “Methods”. The total number of cells in the Q2 and Q4 quadrant were counted as apoptotic cells. **b** Percentages of apoptotic MCF-7-TamR cells are presented in the bar graph. **c** NgBR knockdown decreases cell viability of MCF-7-TamR cells treated with 4-OHT. Cell viability was determined using trypan blue staining. MCF-7-TamR cells were treated with 4-OHT (5 μM) for 48 h. The viable cell number in the non-silencing (NS) group is set as 100%. **d** NgBR knockdown decreases the clonogenenicity of MCF-7-TamR cells treated with 4-OHT (5 μM). Clonogenic survival assay was performed as described in “Methods”. **e** Quantification of colony number in colony formation assays as described in Fig. 2d. The data were repeated in three separate experiments, and are presented as the mean ± SD (*p < 0.05, *n* = 3)

To determine the contribution of increased NgBR expression to tamoxifen resistance, we knocked down the expression of NgBR with specific NgBR siRNA (siNgBR), which has been validated in our previous reports [18]. The effects of NgBR knockdown on cell apoptosis and necrosis of MCF-7-TamR (Fig. 2a and b) and T47D-TamR (Additional file 3: Figure S3A and B) were determined by Annexin V/PI staining and flow cytometry. The results showed that NgBR knockdown increases the sensitivity of both MCF-7-TamR and T47D-TamR cells to 4-OHT. Compared to the non-treatment group, 4-OHT treatment alone did not induce significant death of tamoxifen-resistant cells. However, NgBR knockdown along with 4-OHT treatment significantly increased the percentage of cell death. Cell viability was determined by counting the number of negative trypan blue stained cells using a hemocytometer. Consistently, MCF-7-TamR and T47D-TamR cells were resistant to 4-OHT treatment. However, NgBR knockdown restored the sensitivity of MCF-7-TamR (Fig. 2c) and T47D-TamR (Additional file 3: Figure S3C) to tamoxifen. The clonogenic survival assay further demonstrated that NgBR knockdown attenuates the colony formation capability of MCF-7-TamR (Fig. 2d and e) and T47D-TamR cells (Additional file 3: Figure S3D and E) under the condition of 5 μM 4-OHT treatment. These results demonstrated that NgBR knockdown increases the sensitivity of tamoxifen-resistant ERα-positive breast cancer cells to tamoxifen.

**Fig. 3:**
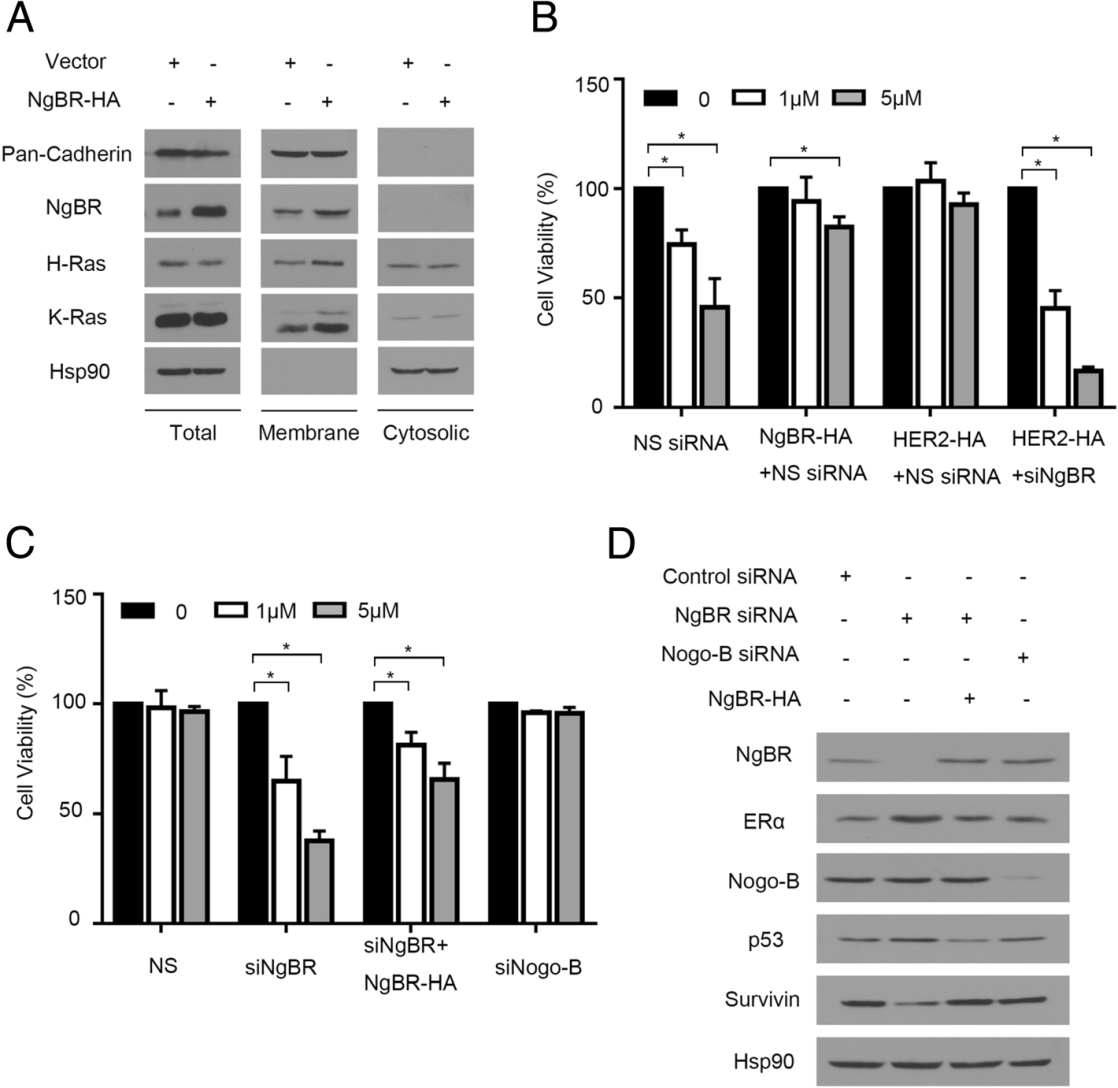
Overexpression of Nogo-B receptor (NgBR) increases the resistance of MCF-7 cells to tamoxifen. **a** Overexpression of NgBR in MCF-7 cells increases the membrane-associated H-Ras and K-Ras. The plasma membrane proteins were isolated by the ultracentrifugation method. Protein levels of pan-cadherin, NgBR, H-Ras, K-Ras and Hsp90 in MCF-7 cells were determined using western blot analysis. **b** Viability of MCF-7 cells treated with 4-OHT (0, 1 or 5 μM) was determined using CCK-8 assay. Overexpression of either human influenza hemagglutinin (HA)-tagged NgBR or HER2 in MCF-7 cells decreases their sensitivity to 4-OHT. Knockdown NgBR in MCF-7 cells restores the sensitivity of MCF-7 cells overexpressing HER2-HA to 4-OHT. The number of viable cells in the untreated group is referred as 100% (*p < 0.05, *n* = 3). **c** Viability of MCF-7-TamR cells treated with 4-OHT (0, 1 or 5 μM) was determined using CCK-8 assay. Knockdown of NgBR in MCF-7-TamR cells increases their sensitivity to 4-OHT. Overexpression of NgBR decreases the sensitivity of NgBR-knockdown MCF-7-TamR cells to 4-OHT. Knockdown of Nogo-B in MCF-7-TamR cells does not affect their sensitivity to 4-OHT. The number of viable cells in the untreated group is referred to as 100% (*p < 0.05, *n* = 3). **d** NgBR regulates the expression of ERα, p53 and survivin independent of its ligand Nogo-B. MCF-7-TamR cells were transfected with control siRNA or NgBR siRNAs targeting either NgBR or Nogo-B. In MCF-7-TamR cells transfected with siRNA targeting the untranslated region of NgBR, NgBR expression was restored by the transfection of NgBR-HA plasmid DNA

As in our previous report [15], overexpression of NgBR in MCF-7 cells increased the membrane-associated H-Ras and K-Ras (Fig. 3a). Consequently, if we transfected plasmid DNA expressing human influenza hemagglutinin (HA) tagged NgBR (NgBR-HA) to MCF-7 cells, we appreciated that the overexpression of exogenous NgBR-HA increased the viability of MCF-7 cells treated with 4-OHT (Fig. 3b). Similarly, overexpression of HER2-HA also increased the resistance of MCF-7 cells to 4-OHT (Fig. 3b). However, NgBR knockdown attenuated the resistance of MCF-7 cells overexpressing HER2-HA (Fig. 3b). In MCF-7-TamR cells, overexpression of NgBR-HA restored the resistance of NgBR knockdown MCF-7-TamR cells to tamoxifen (Fig. 3c). As shown in Fig. 3d, transfection of NgBR-HA plasmid DNA restored the expression of NgBR in MCF-7-TamR cells transfected with NgBR siRNA (siNgBR), which targets the 3′-untranslated region (UTR) of NgBR as described in our previous publication [12]. It suggests that NgBR is one of driving forces for tamoxifen resistance. However, knockdown of Nogo-B does not affect the sensitivity of MCF-7-TamR to tamoxifen (Fig. 3c). The efficacy of Nogo-B siRNA was confirmed by western blot analysis (Fig. 3d).

### NgBR knockdown promotes apoptosis by regulating the expression of p53 and survivin

**Fig. 4:**
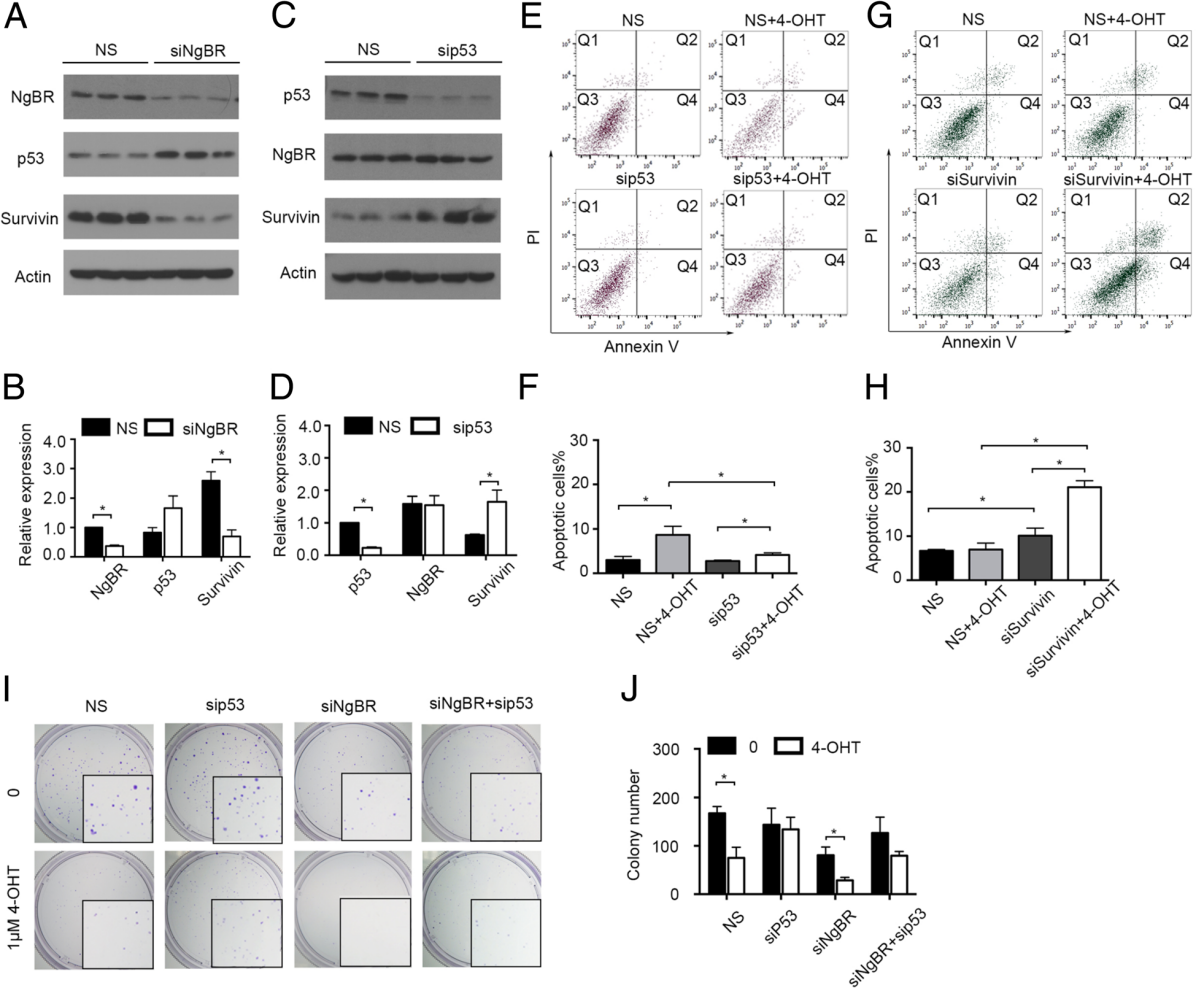
Sensitivity of MCF-7 cells to tamoxifen is regulated by p53 and survivin. **a** Nogo-B receptor (NgBR) knockdown increases p53 protein level and decreases survivin in MCF-7-TamR cells. Protein levels were determined by western blot analysis. **b** Quantitative analyses of proteins presented in Fig. 3a were carried out using ImageJ and were normalized to the housekeeping gene β-actin. Data are presented as fold changes in the siNgBR group compared to the non-silencing (NS) group. **c** Knockdown of p53 increases survivin level in MCF-7 cells. MCF-7 cells were transfected with siRNA specifically targeting p53 as described in “Methods”. The protein levels of p53, NgBR, survivin and β-actin were determined using western blot analysis. **d** Quantitative analyses of proteins presented in Fig. 3c were carried out using ImageJ and were normalized to β-actin. Data are presented as fold changes in the sip53 group compared to the NS group. **e** Knockdown of p53 increases the clonogenenicity of MCF-7 cells. Clonogenic survival assay was used for measuring clonogenicity of MCF-7 cells treated with 4-OHT (1 μM). **f** Quantification of colony number in colony formation assays is presented in Fig. 3e. **g** Survivin knockdown increases apoptosis of MCF-7-TamR cells induced by 4-OHT (5 μM). **h** Percentages of apoptotic cells in Fig. 3g are shown in the bar graph. **i** Knockdown of p53 decreases the sensitivity of NgBR-deficient MCF-7 cells to tamoxifen. NgBR knockdown restored the sensitivity to tamoxifen, which is attenuated by silencing p53. **j** Quantification of colony number in colony formation assays described in Fig. 3i. The data are from three separate repeat experiments, and are presented as the mean ± SD (*p < 0.05, *n* = 3)

**Fig. 5:**
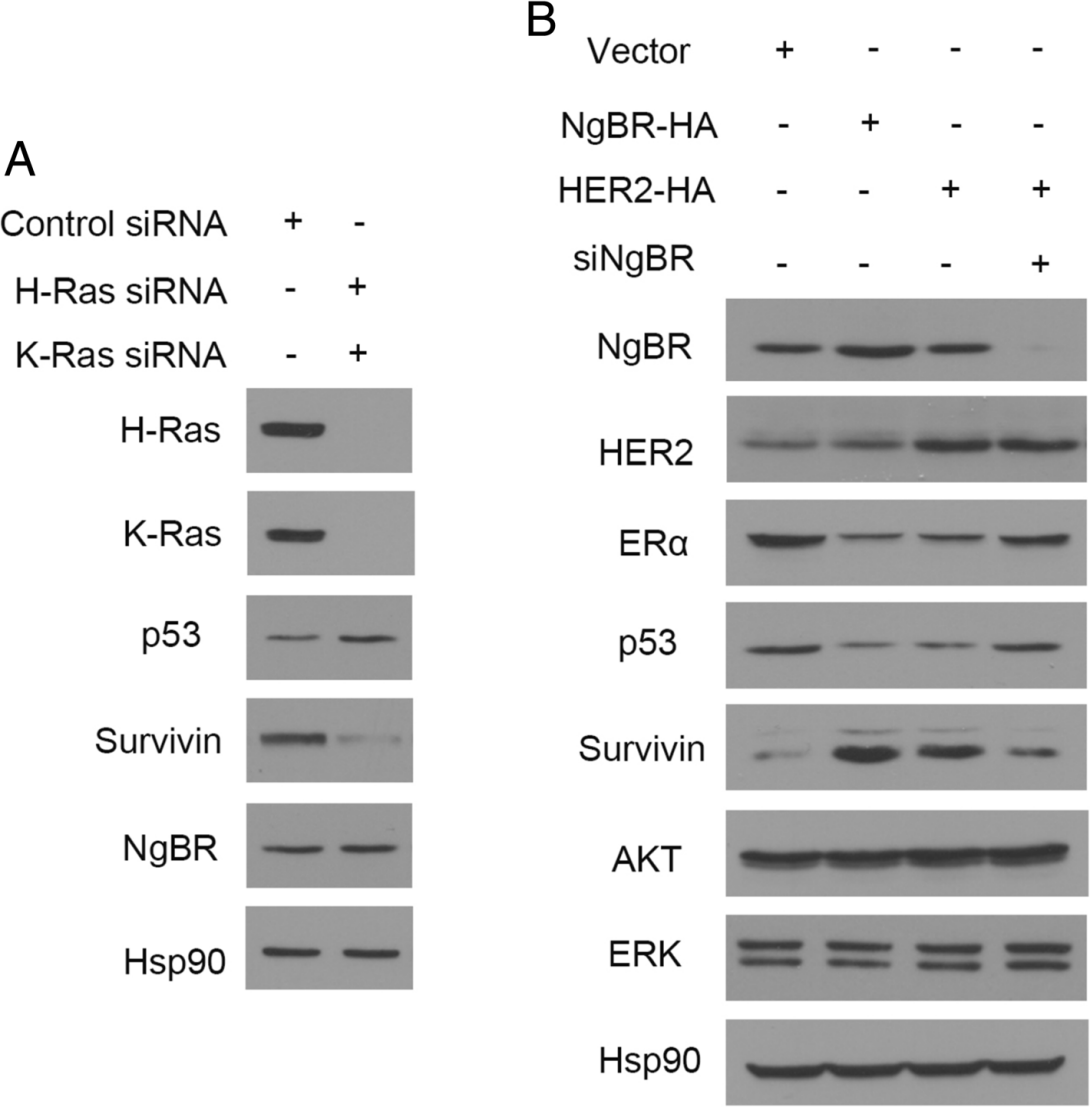
Nogo-B receptor (NgBR) regulates protein levels of p53 and survivin through Ras-mediated pathways. **a** Knockdown of both H-Ras and K-Ras increases the protein levels of p53 and decreases the protein level of survivin. Control siRNA and siRNAs targeting either H-Ras or K-Ras siRNA were transfected into MCF-7-TamR cells. **b** Overexpression of either NgBR-HA or HER2-HA in MCF-7 cells decreases the protein levels of p53, ERα, and increases the protein level of survivin. Knockdown NgBR in MCF-7 cells overexpressing HER2-HA restores the protein levels of p53, ERα and survivin to levels similar to those in control MCF-7 cells transfected with empty plasmid DNA vector. Change in NgBR or human epidermal growth factor receptor 2 (HER2) has no effects on the total protein levels of AKT and extracellular signal-related kinase (ERK)

Our previous study showed that NgBR deficiency in ERα-positive breast cancer cells decreases the resistance to chemotherapy by increasing p53 and decreasing survivin [23]. To determine if and the extent to which NgBR is dependent on p53-mediated survivin expression to promote the resistance to tamoxifen, we examined the alteration of p53 and survivin expression in tamoxifen-resistant ERα-positive breast cancer cells before and after NgBR depletion. As shown in Fig. 4a and b, NgBR depletion in MCF-7-TamR cells increased the expression of p53 but decreased the amount of survivin at the protein levels. Overexpression of NgBR-HA restored the expression pattern of p53 and survivin in MCF-7-TamR cells transfected with siNgBR to levels similar to that in MCF-7-TamR cells transfected with control siRNA (Fig. 3d). When p53 was knocked down by siRNA, survivin was increased in MCF-7 cells (Fig. 4c and d). To determine if either p53 or survivin is involved in regulating the apoptosis of NgBR-deficient cells, we knocked down either p53 in MCF-7 cells or survivin in MCF-7-TamR cells using siRNA either targeting p53 (si-p53) or targeting survivin (si-survivin), respectively. As shown in Fig. 4e and f, knockdown of p53 increased the resistance of MCF-7 cells to 4-OHT, while survivin knockdown restored the sensitivity of MCF-7-TamR cells to 4-OHT (Fig. 4g and h). Similar results were also observed in p53 knockdown T47D cells (Additional file 4: Figure S4A–D) and survivin knockdown T47D-TamR cells (Additional file 4: Figure S4E–F). These results demonstrated the contribution of increased p53 expression and decreased survivin expression that occurs in NgBR knockdown cells for restoring the sensitivity of ERα-positive breast cancer cells to tamoxifen. The clonogenic survival assay further demonstrated that loss of p53 in NgBR knockdown MCF-7 cells attenuated the effects of NgBR deficiency on increased sensitivity of MCF-7 cells to tamoxifen (Fig. 4i and j). In addition, knockdown of both H-Ras and K-Ras in MCF-7-TamR cells also resulted in the increased amount of p53 and decreased amount of survivin (Fig. 5a). Overexpression of either NgBR-HA or HER2-HA in MCF-7 cells decreased the protein levels of p53 and ERα but increased the protein level of survivin (Fig. 5b). Interestingly, knockdown of NgBR in MCF-7 cells overexpressing HER2-HA restored the protein levels of p53, ERα and survivin levels to those occurring in control MCF-7 cells transfected with empty vector (Fig. 5b).

### NgBR knockdown diminished EGF-stimulated phosphorylation of ERα

**Fig. 6:**
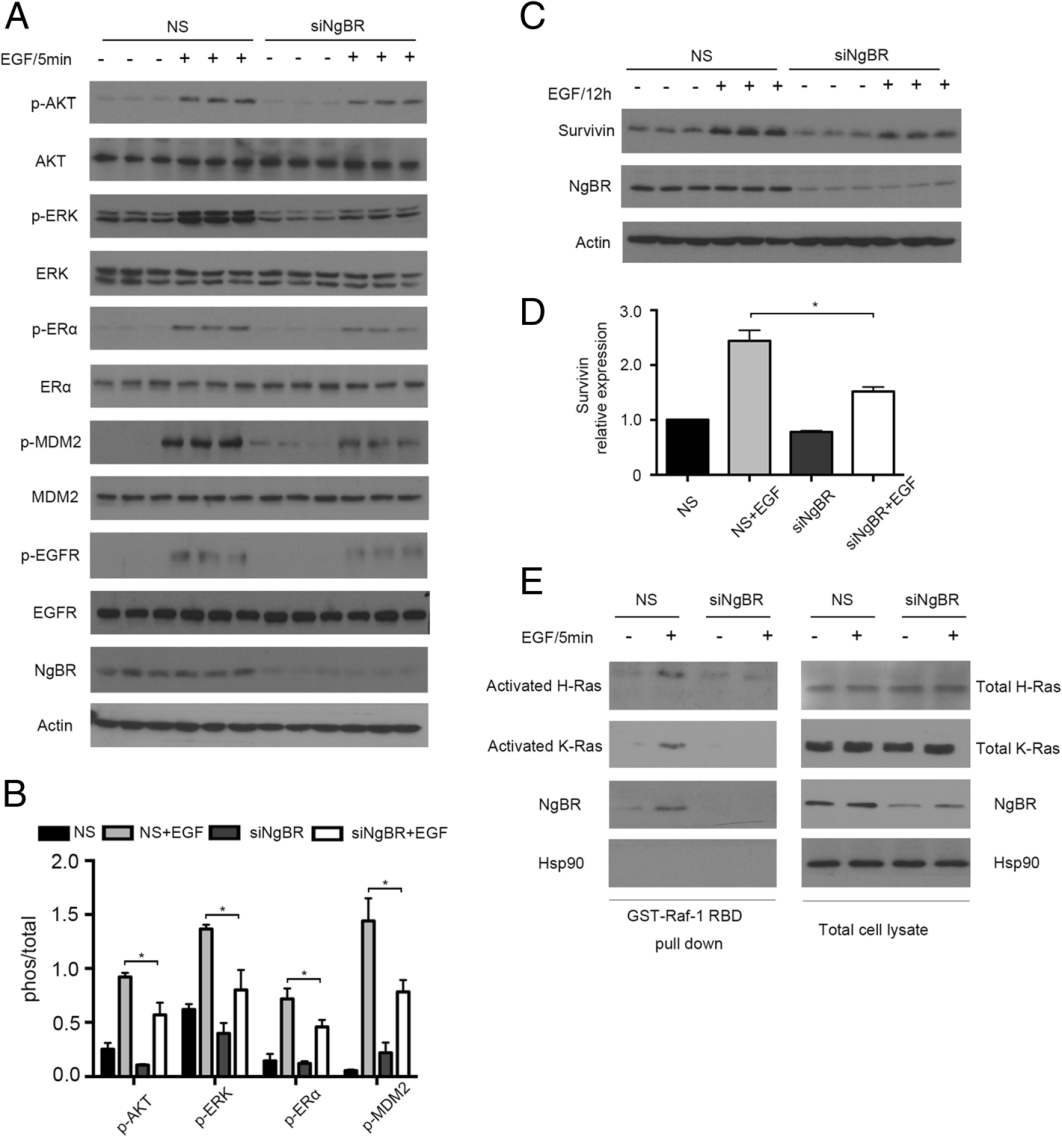
Nogo-B receptor (NgBR) knockdown attenuates epidermal growth factor receptor (EGF)-stimulated signaling and estrogen receptor alpha (ERα) phosphorylation in MCF-7-TamR cells. **a** MCF-7-TamR cells were transfected with siNgBR and treated with EGF (100 ng/mL) for 5 min. Downstream signaling of the EGF pathway was determined using western blot assay. **b** Quantitative analysis of phosphorylated proteins presented in Fig. 4b were carried out using ImageJ and were normalized to total proteins. **c** MCF-7-TamR cells were transfected with siNgBR and treated with 100 ng/mL EGF for 12 h. Survivin protein levels were determined using western blot analysis. **d** Quantitative analysis of survivin protein levels presented in Fig. 4c were carried out using ImageJ and were normalized to β-actin. **e** NgBR is required for the EGF-stimulated activation of H-Ras and K-Ras in MCF-7-TamR cells. MCF-7-TamR cells were transfected with siNgBR and stimulated with 100 ng/mL EGF for 5 min. The complex of activated Ras (GTP-loaded Ras) was precipitated from total cell lysates using GST-RBD beads. Protein levels were detected by western blotting. Both Ras and NgBR were detected in the complexes precipitated by the Raf-pull-down method. The data are from three separate repeat experiments and are presented as the mean ± SD (*p < 0.05, *n* = 3)

Phosphorylation of ERα serine 118 (S118) residue has been reported to be involved in resistance to tamoxifen [24, 25]. To examine the involvement of NgBR in regulating EGF-stimulated phosphorylation of ERα S118, we treated MCF-7-TamR cells with 100 ng/mL EGF for 5 min, which is the peak of the phosphorylation signal in response to EGF stimulation. As shown in Fig. 6a and b, EGF treatment increased the phosphorylation of AKT, ERK and MDM2 in MCF-7-TamR cells treated with control non-silencing (NS) siRNA. NgBR knockdown attenuated EGF-stimulated phosphorylation of AKT, ERK and MDM2. But NgBR knockdown did not affect the total protein levels of AKT, ERK and MDM2 or the phosphorylation of EGFR (Fig. 6a). EGF treatment not only activated the downstream signaling of the EGF pathway, but also increased the phosphorylation of ERα (S118), which is in accordance with previous studies [10, 26]. NgBR knockdown attenuated EGF-stimulated phosphorylation of ERα. The inhibitory effects of NgBR knockdown on EGF-stimulated phosphorylation of ERα were also noted in T47D-TamR cells (Additional file 5: Figure S5).

To further investigate the underlying mechanism by which NgBR regulates the expression of p53 and survivin, we stimulated MCF-7-TamR cells with EGF (100 ng/mL) for 12 h. EGF treatment increased the protein level of survivin, and NgBR knockdown attenuated the protein level of survivin in EGF-treated cells (Fig. 6c and d). To elucidate the roles of NgBR in regulating the EGF-mediated pathway, we used glutathione (GST)-tagged Ras-binding domain of Raf (RBD) to pull down activated Ras as described in our previous publication [15]. As shown in Fig. 6e, EGF stimulation for 5 min not only induced the activation of H-Ras and K-Ras in MCF-7-TamR cells, but also increased the amount of NgBR in the complex of activated K-Ras and H-Ras. It indicates that EGF stimulation increases the association between NgBR and activated Ras. Consistent with our previous reports [15], NgBR knockdown also attenuated the EGF-stimulated Ras activation in MCF-7-TamR cells. These results (Fig. 6) suggest that NgBR-mediated Ras activation may contribute to EGF-stimulated phosphorylation of ERα.

### NgBR expression is associated with survivin and poor survival in patients with breast cancer

**Fig. 7:**
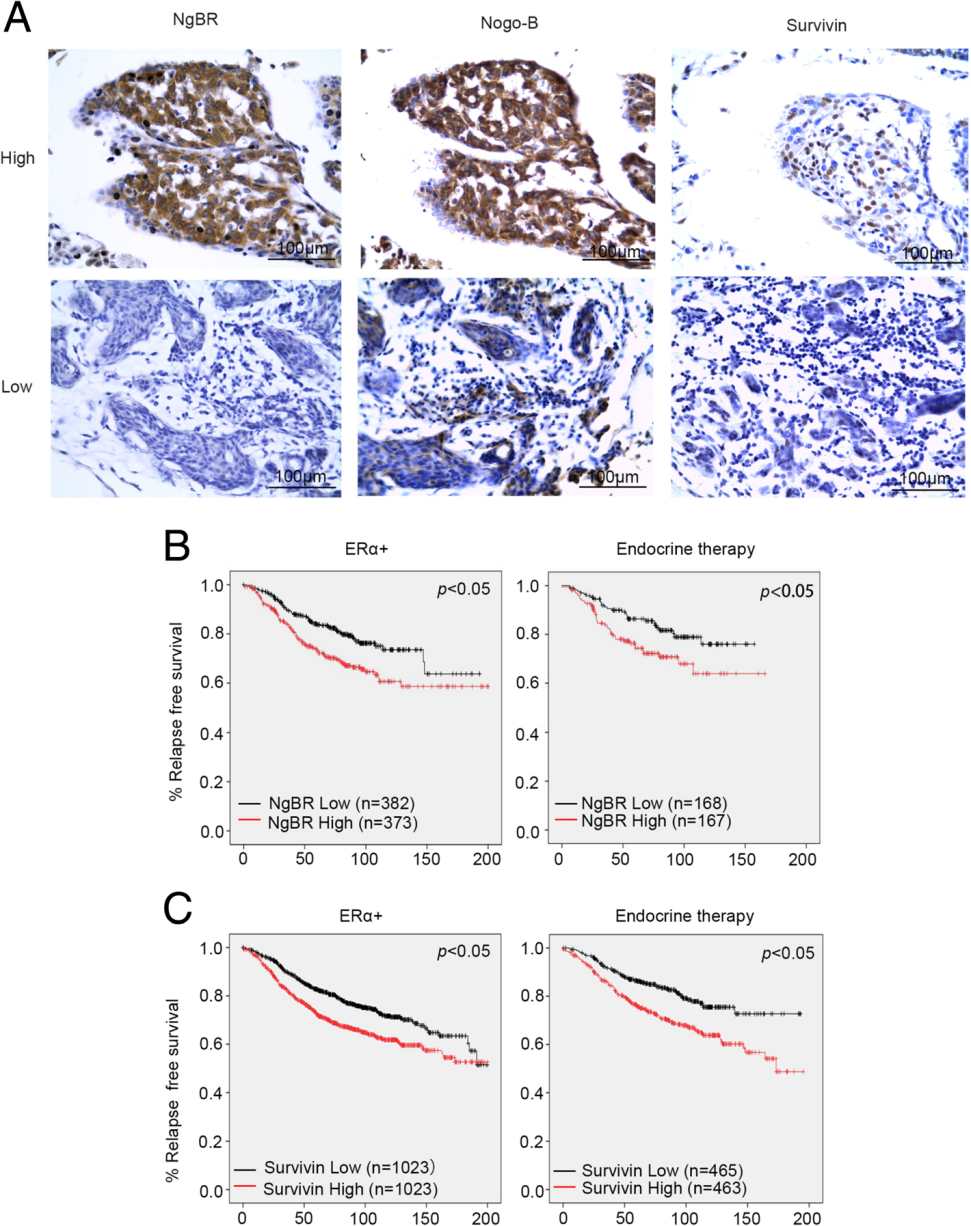
Higher expression of Nogo-B receptor (NgBR) is associated with poor outcome in patients with estrogen receptor alpha (ERα) positive breast cancer. **a** Immunohistocheical (IHC) staining of NgBR, Nogo-B and survivin in 22 samples of breast cancer tissue. Images were taken using an Olympus microscope with × 20 lens. Scale bar 100 μm. **b** Relapse-free survival (RFS) in patients with ERα-positive breast cancer or endocrine therapy-treated patients. NgBR (NUS1) mRNA expression data were retrieved from a gene-expression profiling dataset (225071_x from Kaplan–Meier Plot database) of 755 cases of ERα-positive breast cancer and 335 patients with ERα-positive breast cancer treated with endocrine therapy. Kaplan–Meier analysis revealed significantly reduced RFS (*p* < 0.05) in 373 patients with ERα-positive breast cancer with high NgBR expression in tumors as compared to 382 patients with low NgBR expression in tumors. Similarly, RFS in patients with ERα-positive breast cancer treated with endocrine therapy is significantly decreased in 167 patients with high NgBR expression in tumors as compared to 168 patients with low NgBR expression in tumors (*p* < 0.05). **c** RFS in patients with ERα-positive breast cancer or endocrine therapy-treated patients. Survivin (BIRC5) mRNA expression data were retrieved from a gene-expression profiling dataset (202094_x from Kaplan–Meier Plot database) of 2046 cases of ERα-positive breast cancer and 928 patients with ERα-positive breast cancer treated with endocrine therapy. Kaplan–Meier analysis revealed significantly reduced RFS (*p* < 0.05) in 1023 patients with ERα-positive breast cancer with high survivin expression in tumors as compared to 1023 patients with low survivin expression in tumors. Similarly, RFS in patients with ERα-positive breast cancer treated with endocrine therapy is significantly decreased in 463 patients with high survivin expression in tumors as compared to 465 patients with low NgBR expression in tumors (*p* < 0.05)

Our previous publication showed that expression of NgBR is much higher in ERα-positive breast cancer tissues than in normal breast tissues, and that NgBR is also highly associated with survivin expression [16]. To further confirm the relationship between survivin and NgBR, we performed immunohistochemistry (IHC) staining to examine expression in 22 samples of breast cancer tissue. The basic characteristics of the tissue samples are shown in Table 1, and the results of the quantitative analysis of IHC staining was shown in Table 2. As shown in Fig. 7a, patients with high expression of NgBR also have high expression of survivin. In patients with negative or weak expression of NgBR, the expression of survivin was also low. The association between NgBR and survivin was statistically significant (Table 2). The association between NgBR and clinical outcomes in patients with breast cancer was determined using the public Kaplan–Meier Plot database. Kaplan–Meier analysis revealed that high expression of NgBR was associated with poor RFS in patients with ERα-positive breast cancer (*n* = 755) and in patients receiving endocrine therapy (*n* = 335) (Fig. 7b; Additional files 6, 7, 8 and 9). Consistently, high expression of survivin was also associated with poor RFS in patients with ERα-positive breast cancer (*n* = 2046) and in patients receiving endocrine therapy (*n* = 928) (Fig. 7c). The association between higher NgBR expression and poor RFS was further confirmed in the GSE6532 dataset (*n* = 343) (Additional file 10: Figure S6; Additional file 11).

**Table 1:** Demographic and clinical characteristics of study population

	Variables	Value
ER	1+	1 (0.05)
	2+	9 (0.41)
	3+	12 (0.54)
PR	1+	4 (0.18)
	2+	5 (0.23)
	3+	13 (0.59)
HER2	–	17 (0.77)
	1+	2 (0.09)
	2+	2 (0.09)
	3+	1 (0.05)

*Abbreviations*: *ER* estrogen receptor, *PR* progesterone receptor, *HER2* human epidermal growth factor receptor 2

**Table 2:** Correlation analysis of survivin and NgBR

	Survivin		Number	*p*
	Low	High		
NgBR				
Low	5 (71.4%)	2 (28.6%)	7 (100%)	0.014
High	2 (13.3%)	13 (86.7%)	15 (100%)	
*n*	7 (31.8%)	15 (68.2%)	22 (100%)	

*NgBR* Nogo-B receptor

## Discussion

As previously confirmed, NgBR is highly expressed in ERα-positive breast cancer [16], and promotes epithelial-mesenchymal transition of breast tumor cells [18]. However, the underlying mechanism by which NgBR enhances the acquired resistance of ERα-positive breast cancer to tamoxifen has not been elucidated. In this study, we found that NgBR expression is increased in tamoxifen-resistant breast cancer cell lines (Fig. 1). High expression of NgBR was associated with poor RFS in patients with ERα-positive breast cancer and in patients receiving endocrine therapy (Fig. 5b). NgBR knockdown decreased EGF-induced expression of survivin (Fig. 4c and d) and phosphorylation of ERα (Fig. 4a). Consequently, the results of cell viability and apoptosis assays clearly demonstrate that NgBR knockdown attenuates resistance to tamoxifen (Fig. 2). Our study elucidated the important roles of NgBR in promoting the acquired resistance of ERα-positive breast cancer to tamoxifen.

For patients with ERα positive breast cancer, treatment mainly focuses on reducing estrogen levels or blocking the ERα signaling pathway. Aromatase inhibitors (AIs), such as anastrozole [27], are estrogen synthesis inhibitors. Fulvestrant is a selective ERα downregulator [28]. Tamoxifen, also known as a selective estrogen receptor modulator, blocks the activity of estrogen by binding to ERα [29] and suppressing the classical ERE regulated genes [30]. However, 30% of patients still gain resistance to tamoxifen [31]. Many studies have elucidated potential mechanisms of tamoxifen resistance, but these are still unclear due to many unidentified factors [32]. In this study, we demonstrated that NgBR, which is upregulated in tamoxifen-resistant breast cancer cells, is a potential factor contributing to tamoxifen resistance. Our data demonstrated that NgBR knockdown restores the sensitivity of tamoxifen-resistant breast cancer cells to tamoxifen (Fig. 2). Our result indicates NgBR is a potential therapeutic target for attenuating tamoxifen resistance. Unlike NgBR, Nogo-B protein levels do not increase in either MCF-7-TamR cells (Fig. 1g) or T47D-TamR cells (Additional file 2: Figure S2). Although NgBR was identified as a specific receptor for ligand Nogo-B, knockdown of Nogo-B does not affect the sensitivity of MCF-7-TamR cells to tamoxifen (Fig. 3c). If and the extent to which Nogo-B facilitates the NgBR-mediated Ras-signaling pathway needs further investigation.

According to previous studies, increased EGFR expression in tamoxifen-resistant cells contributes to the acquired resistance to tamoxifen [10, 21]. Consistent with previous reports, EGF stimulation also activated the phosphorylation of ERα [33]. Our recent report demonstrated that NgBR binds the farnesylated Ras and promotes Ras plasma membrane translocation [15]. NgBR-mediated accumulation of plasma membrane-associated Ras enhances EGF signaling [15]. As shown in Fig. 3a, NgBR overexpression in MCF-7 cells also increased membrane-associated H-Ras and K-Ras and resulted in increased resistance to tamoxifen (Fig. 3b). Similarly, increased expression of NgBR in tamoxifen-resistant breast cancer cells also enhances EGF-stimulated Ras activation and phosphorylation of AKT and ERK. NgBR knockdown diminishes the EGF-stimulated Ras activation and EGFR-mediated signaling (Fig. 6). Activation of these pathways leads to Akt-dependent phosphorylation of MDM2 [34], which downregulates cellular levels of p53 and decreases p53 transcriptional activity [35]. Our previous report also demonstrated that NgBR promotes the ubiquitination of p53 in human hepatocellular carcinoma via Akt and MDM2 phosphorylation signaling [36]. Here, our data demonstrated the inverse expression patterns between p53 and NgBR in breast cancer cells (Fig. 4). The protein levels of p53 decrease in tamoxifen-resistant breast cancer cells along with increased expression of survivin and NgBR (Fig. 1g; Additional file 2: Figure S2A). Knockdown of either NgBR (Fig. 3d) or H-Ras/K-Ras (Fig. 5a) in MCF-7-TamR cells increased p53 and deceased survivin protein levels. As a tumor repressor gene, p53 is found to be mutated in many cancers [37] and promotes apoptosis in breast cancers [38]. It has been shown that p53 represses survivin expression at the transcriptional level [39]. Our data (Fig. 4c and d; Additional file 4: Figure S4A and B) also demonstrated that knockdown of p53 in breast cancer cells can induce the expression of survivin, which predicts a poor response to endocrine therapy [40]. In this study, we confirmed the effects of p53 on inducing tamoxifen resistance in parental MCF-7 and T47D cells, and survivin knockdown restored the sensitivity to 4-OHT in MCF-7-TamR (Fig. 4) and T47D-TamR cells (Additional file 4: Figure S4).

Ras is a well-known oncogene that has been shown to cause tumorigenesis and drug resistance by activating downstream kinases such as phosphatidylinositol-3-OH kinase (PI-3 K)/Akt and Raf-1 kinase/ERK [41–45]. Although Ras mutations rarely occur in breast cancer (less than 10%) [46], oncogenic Ras can contribute to the tumorigenic and invasive potential of breast epithelial cells [46]. Therefore, upregulation of normal Ras activity by RTKs, such as the EGFR and insulin growth factor receptor (IGF1-R), has been shown in ERα positive breast cancer [47–49]. The classic mechanism of E2 action is mediated by the nuclear ERα that regulates transcription of target genes containing the consensus ERE in their promoter region [47–49]. In addition, E2 can also exert its action through membrane ERα (mERα) in conjugation with the signaling complex including EGFR, IGF-1R, adaptor protein Shc/Grb2 and RasGEFs (such as SOS1 and RasGEF3) to activate Src/Ras-dependent activation of the Raf1-MARK/PI3K-Akt pathways [47–49]. This pathway promotes estrogen-dependent tumor resistance [50]. Our recent publication demonstrated that NgBR binds farnesylated Ras and is required for keeping Ras at the plasma membrane [15]. Therefore, NgBR is essential for the Ras-mediated signaling pathway [15]. Previous reports have shown that EGF induces the phosphorylation of ERα at the serine 118 residue [51], which is the confirmed signal involved in ERα-mediated resistance to endocrine therapy [25]. In this study, we demonstrated that NgBR knockdown impairs EGF-stimulated phosphorylation of ERα (Fig. 4a; Additional file 10: Figure S6A) but also attenuates resistance to tamoxifen (Fig. 2; Additional file 3: Figure S3). This finding indicates that NgBR is a potential therapeutic target for blocking concurrent endocrine-resistant signaling. However, we need further investigation to determine synergetic roles of NgBR in coordinating with other growth factor receptors, such as insulin-like growth factor 1 receptor (IGF1-R) and mERα, to promote the acquired resistance to tamoxifen.

Except for the contribution of growth factor receptor signaling pathways to tamoxifen resistance [9, 10], decreased ERα expression [7, 52] and increased human epidermal growth factor receptor 2 (HER2) [8, 53] are more likely to be attributed to tamoxifen resistance. Previous studies by others showed that hyperactivation of Raf kinase induces the loss of ERα in ERα-positive breast cancer cells [54] and inhibition of mitogen-activated protein kinase (MAPK) activity induced the expression of ERα in ERα-negative breast cancer cells [55]. As shown in Fig. 1g, ERα decreased and HER2 increased in MCF-7-TamR cells as compared to parental MCF-7 cells. Overexpression of either NgBR-HA or HER2-HA in MCF-7 cells decreased the protein level of ERα (Fig. 5b). Interestingly, NgBR knockdown in either MCF-7-TamR cells (Fig. 3d and c) or MCF-7 cells overexpressing HER2-HA (Figs. 5b and 3b) restored the protein level of ERα to a level similar to that in control cells as well as sensitivity to tamoxifen. The contribution of NgBR-regulated ERα expression to tamoxifen resistance needs further investigation in our future studies.

## Conclusions

**Fig. 8:**
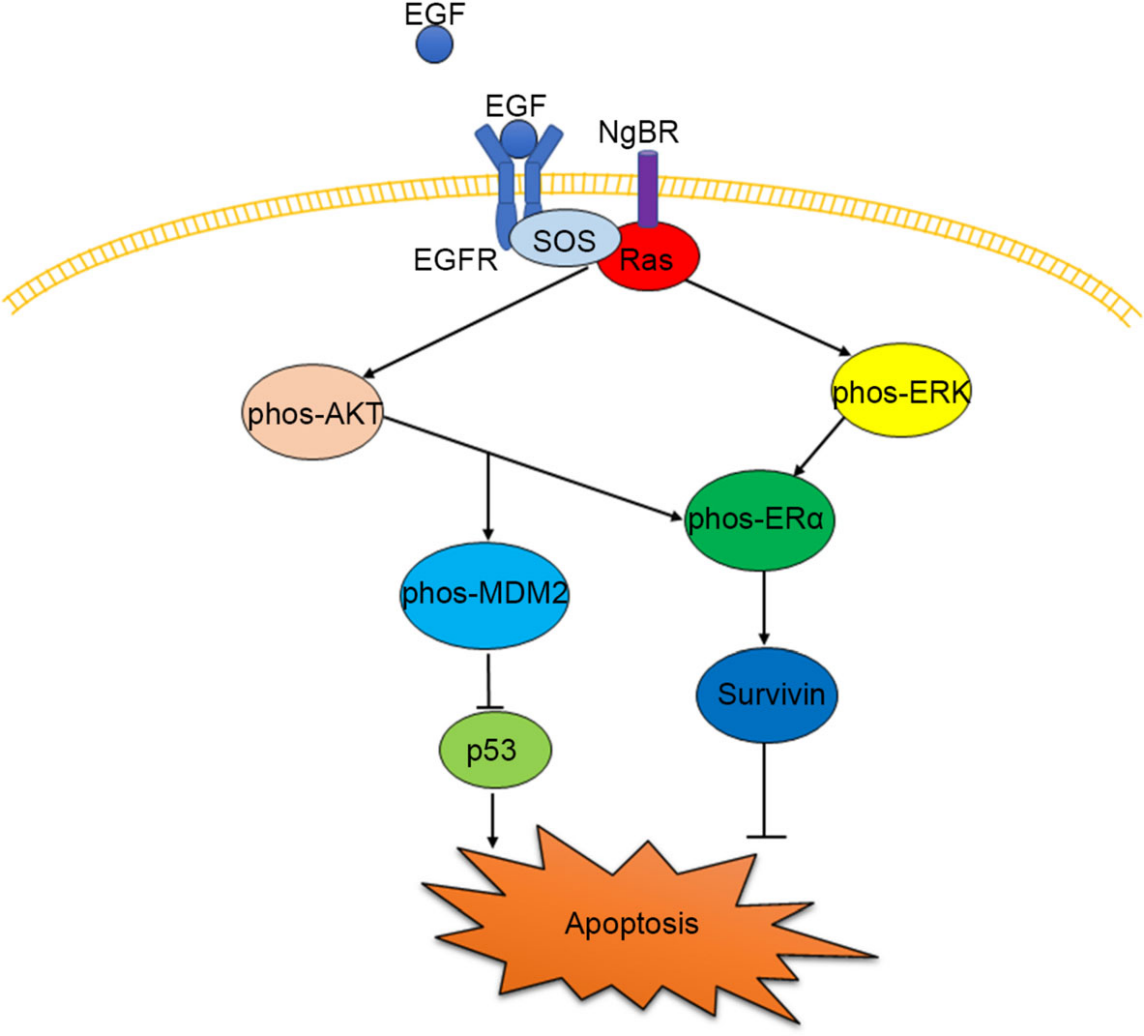
Nogo-B receptor (NgBR) is required for epidermal growth factor (EGF)-acquired resistance to tamoxifen in patients with estrogen receptor alpha (ERα)-positive breast cancer. EGF binds to EGF receptor (EGFR) and recruits SOS1, an activator for Ras, to the plasma membrane. NgBR promotes the translocation of Ras to the plasma membrane and enhances EGF signaling, such as phosphorylation of Akt and extracellular signal-related kinase (ERK). Phosphorylated Akt further induces the phosphorylation of MDM2, which promotes p53 ubiquitination leading to its degradation and attenuates p53-mediated apoptosis. Phosphorylation of Akt and ERK also induce the phosphorylation of ERα, which induces the expression of survivin, an apoptosis inhibitor

In summary, our results demonstrate that increased expression of NgBR in tamoxifen-resistant breast cancer cells promotes EGF signaling by increasing phosphorylation of AKT/ERK and MDM2, which attenuates the expression of p53 and increases the expression of survivin, which may lead to acquired resistance to tamoxifen as shown in Fig. 8. Higher levels of NgBR in patients with ERα-positive breast cancer are associated with poor RFS outcomes in patients with breast cancer because they lead to easier acquisition of tamoxifen resistance. Our data suggest that NgBR is a potential therapeutic target for restoring the sensitivity of tamoxifen-resistant breast cancer cells to conventional endocrine therapy.

## Additional files

Additional file 1: **Figure S1.** MCF-7-TamR and T47D-TamR cells are resistant to 4-OHT. The 4-OHT resistant phenotype was confirmed using the CCK8 cell viability assay. (A) Cell viability was analyzed in MCF-7 and MCF-7-TamR cells treated with 1 μM 4OHT for different time periods (0, 1 day, 3 days and 5 days). (B) Cell viability was analyzed in T47D and T47D-TamR cells treated with 1 μM 4OHT for different time periods (0, 1 day, 3 days and 5 days). The OD value of untreated cells is referred to as 100%. The results show the average percentage of OD value as compared to untreated cells. The data are from in three separate repeated experiments, and are presented as the mean ± SD (*p < 0.05, *n* = 3). (PDF 153 kb)

Additional file 2: **Figure S2.** NgBR is highly expressed in the tamoxifen-resistant T47D-TamR cells. (A) NgBR level was increased in T47D-TamR cells. Protein levels of Nogo-B, ERα, p53 and survivin in T47D and T47D-TamR cells were determined using western blot analysis. (B) Quantitative analysis of proteins presented in Additional file 2: Figure S2A was carried out using ImageJ and normalized to β-actin. Data are presented as fold changes of T47D-TamR compared to the T47D cells. The data are from three separate repeated experiments and are presented as the mean ± SD (*p < 0.05, *n* = 3). (PDF 155 kb)

Additional file 3: **Figure S3.** NgBR decreases the resistance of T47D-TamR to tamoxifen. (A) NgBR knockdown increases apoptosis of T47D-TamR cells induced by 4-OHT (5 μM). The apoptotic cells were detected by Annexin V-PI staining. The total number of cells in the Q2 and Q4 quadrant was regarded as apoptotic cells. (B) Percentages of apoptotic cells are shown in the bar graph. (C) NgBR knockdown decreases the viability of T47D-TamR cells. Cell viability was determined using trypan blue staining of T47D-TamR cells treated with 5 μM 4-OHT for 48 h. The viable cell number of the NS group is referred to as 100%. (D) NgBR knockdown decreases the clonogenenicity of T47D-TamR cells. The clonogenic survival assay was used for measuring clonogenicity of T47D-TamR cells treated with 4-OHT (5 μM). (E) Quantification of colony number in colony formation assays presented in Additional file 3: Figure S3D. The data are from three separate repeated experiments and are presented as the mean ± SD (*p < 0.05, *n* = 3). (PDF 296 kb)

Additional file 4: **Figure S4.** Sensitivity of T47D cells to tamoxifen is regulated by p53 and survivin (A) Knockdown of p53 increases survivin level in T47D cells. T47D cells were transfected with siRNA specifically targeting p53 as described in “Methods”. The protein levels of p53, NgBR, survivin and β-actin were determined using western blot analysis. (B) Quantitative analysis of proteins presented in Additional file 5: Figure S5A were carried out using ImageJ and were normalized to β-actin. Data are presented as fold changes in the si-p53 group compared to the NS group. (C) Knockdown of p53 decreases apoptosis of T47D cells induced by 4-OHT (1 μM). (D) Percentages of apoptotic cells in Additional file 5: Figure S5C are shown in the bar graph. (E) Survivin knockdown increases apoptosis of T47D-TamR cells induced by 4-OHT (5 μM). (F) Percentages of apoptotic cells in Additional file 5: Figure S5E are shown in the bar graph. (PDF 292 kb)

Additional file 5: **Figure S5.** NgBR knockdown attenuated EGF-stimulated signaling and ERα phosphorylation in T47D-TamR cells. (A) T47D-TamR cells were transfected with siNgBR and treated with EGF (100 ng/mL) for 5 min. Downstream signaling of the EGF pathway was determined using western blot assay. (B) Quantitative analysis of phosphorylated proteins presented in Additional file 10: Figure S6A were carried out using ImageJ and were normalized to total proteins. The data are from three separate repeated experiments and are presented as the mean ± SD (*p < 0.05, n = 3). (PDF 310 kb)

Additional file 6: NgBR (NUS1) mRNA expression data were retrieved from a gene-expression profiling dataset (225071_x from Kaplan–Meier Plot database) of 755 patients with ERα-positive breast cancer and 335 patients with ERα-positive breast cancer treated with endocrine therapy. Kaplan–Meier analysis revealed significantly reduced relapse-free survival (RFS) (*p* < 0.05) in 373 patients with ERα positive breast cancer with high NgBR expression in tumors as compared to 382 patients with low NgBR expression in tumors. (XLSX 42 kb)

Additional file 7: NgBR (NUS1) mRNA expression data were retrieved from a gene-expression profiling dataset (225071_x from Kaplan–Meier Plot database) of 755 patients with ERα-positive breast cancer and 335 patients with ERα-positive breast cancer treated with endocrine therapy. Kaplan–Meier analysis revealed that the RFS in patients with ERα-positive breast cancer treated with endocrine therapy is significantly decreased in 167 patients with high NgBR expression in tumors as compared to 168 patients with low NgBR expression in tumors (*p* < 0.05). (XLSX 32 kb)

Additional file 8: Survivin (BIRC5) mRNA expression data were retrieved from a gene-expression profiling dataset (202094_x from Kaplan–Meier Plot database) of 2046 patients with ERα-positive breast cancer and 928 patients with ERα-positive breast cancer treated with endocrine therapy. Kaplan–Meier analysis revealed significantly reduced relapse-free survival (RFS) (*p* < 0.05) in 1023 patients with ERα-positive breast cancer with high survivin expression in tumors as compared to 1023 patients with low survivin expression in tumors. (XLSX 70 kb)

Additional file 9: Survivin (BIRC5) mRNA expression data were retrieved from a gene-expression profiling dataset (202094_x from Kaplan–Meier Plot database) of 2046 patients with ERα-positive breast cancer and 928 patients with ERα-positive breast cancer treated with endocrine therapy. Kaplan–Meier analysis revealed that RFS in patients with ERα-positive breast cancer treated with endocrine therapy is significantly decreased in 463 patients with high survivin expression in tumors as compared to 465 patients with low NgBR expression in tumors (*p* < 0.05). (XLSX 60 kb)

Additional file 10: **Figure S6.** Relapse-free survival (RFS) in patients with ERα-positive breast cancer (*n* = 343). NgBR (NUS1) mRNA expression data were retrieved from the GSE6532 database. Kaplan–Meier analysis revealed significantly reduced RFS (*p* < 0.05) in patients with high NgBR expression in tumors (*n* = 189) as compared to patients with low NgBR expression in tumors (*n* = 154). (PDF 109 kb)

Additional file 11: NgBR (NUS1) mRNA expression data were retrieved from GSE6532 database. Kaplan–Meier analysis revealed significantly reduced relapse-free survival (RFS) (*p* < 0.05) in patients with high NgBR expression in tumors (*n* = 189) as compared to patients with low NgBR expression in tumors (*n* = 154). (XLS 67 kb)

## Abbreviations

4-OHT: 4-Hydroxytamoxifen
AIs: Aromatase inhibitors
AmNogo-B: Amino terminus of Nogo-B
DFS: Disease-free survival
DMEM: Dulbecco’s modified Eagle’s medium
EGF: Epidermal growth factor
EGFR: Epidermal growth factor receptor
EMT: Epithelial-mesenchymal transition
ERα: Estrogen receptor alpha
ERK: Extracellular signal-related kinase
FBS: Fetal bovine serum
GST: Glutathione
HA: Human influenza hemagglutinin
HER2: Human epidermal growth factor receptor 2
IHC: Immunohistochemistry
MAPK: Mitogen-activated protein kinase
MCF-7-TamR: Tamoxifen resistant MCF-7 cells
mERα: Membrane ERα
mRNA: Messenger RNA
NCCN: National Comprehensive Cancer Network
NgBR: Nogo-B receptor
NgBR-HA: Human influenza hemagglutinin (HA) tagged NgBR
NS: Non-silencing small interfering RNA
OS: Overall survival
PBS: Phosphate-buffered saline
PR: progesterone receptor
RBD: Ras-binding domain of Raf
RFS: Relapse-free survival
RTKs: Receptor tyrosine kinases
SERM: Selective estrogen receptor modulator
siNgBR: Nogo-B receptor small interfering RNA
siRNA: small interfering RNA
T47D-TamR: Tamoxifen-resistant T47D cells
