# Supplementary material for: Nogo-B receptor increases the resistance to tamoxifen in estrogen receptor-positive breast cancer cells
Source: Breast Cancer Res. 2018 Sep 12;20:112. doi: 10.1186/s13058-018-1028-5 (PMC6134690; doi:10.1186/s13058-018-1028-5)
Supplement: Supplementary file 1 — Figure S1. MCF-7-TamR and T47D-TamR cells are resistant to 4-OHT. The 4-OHT resistant phenotype was confirmed using the CCK8 cell viability assay. (A) Cell viability was analyzed in MCF-7 and MCF-7-TamR cells treated with 1 μM 4OHT for different time periods (0, 1 day, 3 days and 5 days). (B) Cell viability was analyzed in T47D and T47D-TamR cells treated with 1 μM 4OHT for different time periods (0, 1 day, 3 days and 5 days). The OD value of untreated cells is referred to as 100%. The results show the average percentage of OD value as compared to untreated cells. The data are from in three separate repeated experiments, and are presented as the mean ± SD (*p < 0.05, n = 3). (PDF 153 kb) [file 13058_2018_1028_MOESM1_ESM.pdf]

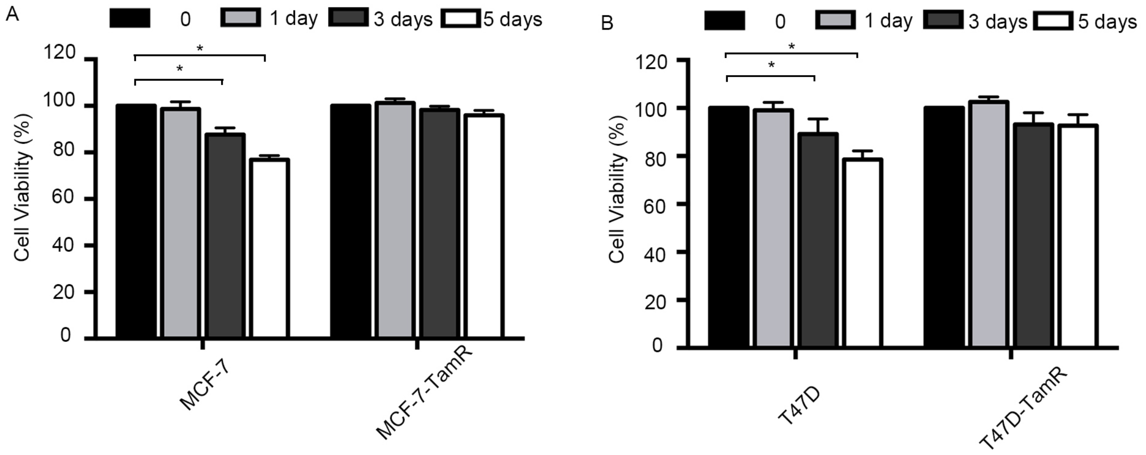

Figure S1 MCF-7-TamR and T47D-TamR cells are resistant to 4-OHT. The 4-OHT resistant phenotype was confirmed using CCK8 cell viability assay. (A) Cell viability was analyzed in MCF-7 and MCF-7-TamR cells treated with 1 μM 4OHT for different time periods (0, 1 day, 3 days and 5 days). (B) Cell viability was analyzed in T47D and T47D-TamR cells treated with 1 μM 4OHT for different time periods (0, 1 day, 3 days and 5 days). The OD value of untreated cells is referred as 100%. The results show the average percentage of OD value as compared to untreated cells. The data were repeated in three separate experiments, and are presented as the mean ± SD. (* p<0.05, n=3).
