# Supplementary material for: Nogo-B receptor increases the resistance to tamoxifen in estrogen receptor-positive breast cancer cells
Source: Breast Cancer Res. 2018 Sep 12;20:112. doi: 10.1186/s13058-018-1028-5 (PMC6134690; doi:10.1186/s13058-018-1028-5)
Supplement: Supplementary file 2 — Figure S2. NgBR is highly expressed in the tamoxifen-resistant T47D-TamR cells. (A) NgBR level was increased in T47D-TamR cells. Protein levels of Nogo-B, ERα, p53 and survivin in T47D and T47D-TamR cells were determined using western blot analysis. (B) Quantitative analysis of proteins presented in Additional file 2: Figure S2A was carried out using ImageJ and normalized to β-actin. Data are presented as fold changes of T47D-TamR compared to the T47D cells. The data are from three separate repeated experiments and are presented as the mean ± SD (*p < 0.05, n = 3). (PDF 155 kb) [file 13058_2018_1028_MOESM2_ESM.pdf]

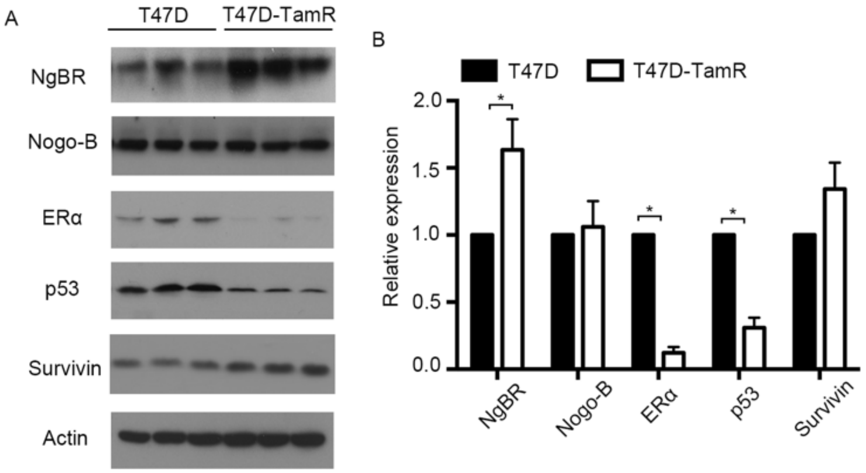


**Figure S2 NgBR is highly expressed in the tamoxifen resistant T47D-TamR cells.** (A) NgBR level was increased in T47D-TamR cells. Protein levels of Nogo-B, ERα, p53 and survivin in T47D and T47D-TamR cells were determined using Western blot analysis. (B) Quantitative analysis of proteins presented in Figure S2A was carried out by using ImageJ and being normalized to β-actin. Data is presented as fold changes of T47D-TamR compared to the T47D cells. The data were repeated in three separate experiments, and are presented as the mean ± SD. (* $p$<0.05, n=3).
