# Supplementary material for: Nogo-B receptor increases the resistance to tamoxifen in estrogen receptor-positive breast cancer cells
Source: Breast Cancer Res. 2018 Sep 12;20:112. doi: 10.1186/s13058-018-1028-5 (PMC6134690; doi:10.1186/s13058-018-1028-5)
Supplement: Supplementary file 3 — Figure S3. NgBR decreases the resistance of T47D-TamR to tamoxifen. (A) NgBR knockdown increases apoptosis of T47D-TamR cells induced by 4-OHT (5 μM). The apoptotic cells were detected by Annexin V-PI staining. The total number of cells in the Q2 and Q4 quadrant was regarded as apoptotic cells. (B) Percentages of apoptotic cells are shown in the bar graph. (C) NgBR knockdown decreases the viability of T47D-TamR cells. Cell viability was determined using trypan blue staining of T47D-TamR cells treated with 5 μM 4-OHT for 48 h. The viable cell number of the NS group is referred to as 100%. (D) NgBR knockdown decreases the clonogenenicity of T47D-TamR cells. The clonogenic survival assay was used for measuring clonogenicity of T47D-TamR cells treated with 4-OHT (5 μM). (E) Quantification of colony number in colony formation assays presented in Additional file 3: Figure S3D. The data are from three separate repeated experiments and are presented as the mean ± SD (*p < 0.05, n = 3). (PDF 296 kb) [file 13058_2018_1028_MOESM3_ESM.pdf]

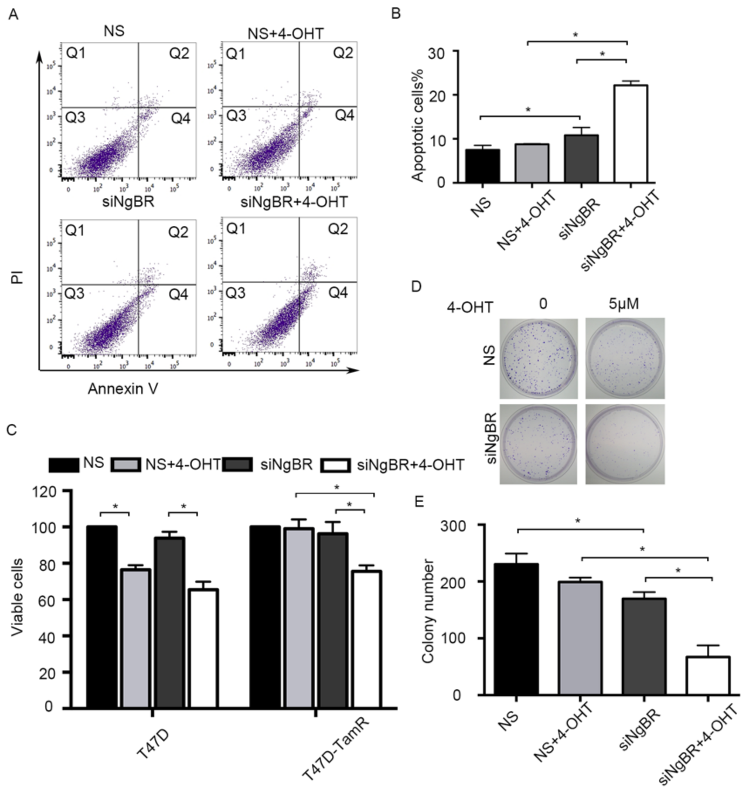

Figure S3 NgBR decreases the resistance of T47D-TamR to tamoxifen. (A) NgBR knockdown increases apoptosis of T47D-TamR cells induced by 4-OHT (5μM). The apoptotic cells were detected by Annexin V-PI staining. The total number of cells in the Q2 and Q4 quadrant was regarded as apoptotic cells. (B) Percentages of apoptotic cells are shown in the bar graph. (C) NgBR knockdown decreases the viability of T47D-TamR cells. Cell viability was determined using trypan blue staining of T47D-TamR cells treated with 5μM 4-OHT for 48h. The viable cell number of NS group is referred as 100%. (D) NgBR knockdown decreases the clonogenenicity of T47D-TamR cells. Clonogenic survival assay was used for measuring clonogenicity of T47D-TamR cells treated with 4-OHT (5μM). (E) Quantification of colony number in colony formation assays presented in figure S3D. The data were repeated in three separate experiments, and are presented as the mean ± SD. (* p<0.05, n=3).
