# Supplementary material for: Nogo-B receptor increases the resistance to tamoxifen in estrogen receptor-positive breast cancer cells
Source: Breast Cancer Res. 2018 Sep 12;20:112. doi: 10.1186/s13058-018-1028-5 (PMC6134690; doi:10.1186/s13058-018-1028-5)
Supplement: Supplementary file 4 — Figure S4. Sensitivity of T47D cells to tamoxifen is regulated by p53 and survivin (A) Knockdown of p53 increases survivin level in T47D cells. T47D cells were transfected with siRNA specifically targeting p53 as described in “Methods”. The protein levels of p53, NgBR, survivin and β-actin were determined using western blot analysis. (B) Quantitative analysis of proteins presented in Additional file 5: Figure S5A were carried out using ImageJ and were normalized to β-actin. Data are presented as fold changes in the si-p53 group compared to the NS group. (C) Knockdown of p53 decreases apoptosis of T47D cells induced by 4-OHT (1 μM). (D) Percentages of apoptotic cells in Additional file 5: Figure S5C are shown in the bar graph. (E) Survivin knockdown increases apoptosis of T47D-TamR cells induced by 4-OHT (5 μM). (F) Percentages of apoptotic cells in Additional file 5: Figure S5E are shown in the bar graph. (PDF 292 kb) [file 13058_2018_1028_MOESM4_ESM.pdf]

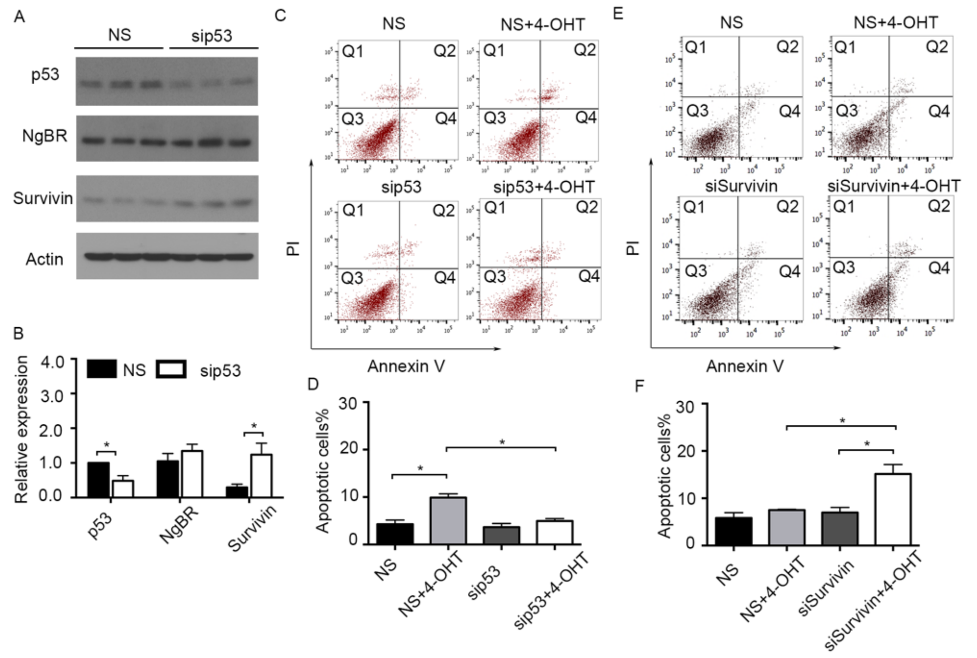

Figure S4 Sensitivity of T47D cells to tamoxifen is regulated by p53 and survivin (A) Knockdown of p53 increases survivin level in T47D cells. T47D cells were transfected with siRNA specifically targeting p53 as described in the Methods. The protein levels of p53, NgBR, survivin and β-actin were determined using Western blot analysis. (B) Quantitative analysis of proteins presented in Figure S5A were carried out using ImageJ and were normalized to β-actin. Data is presented as fold changes of si-p53 group compared to the NS group. (C) Knockdown of p53 decreases apoptosis of T47D cells induced by 4-OHT (1μM). (D) Percentages of apoptotic cells in Figure S5C are shown in the bar graph. (E) Survivin knockdown increases apoptosis of T47D-TamR cells induced by 4-OHT (5μM). (F) Percentages of apoptotic cells in Figure S5E are shown in the bar graph.
