# Supplementary material for: Nogo-B receptor increases the resistance to tamoxifen in estrogen receptor-positive breast cancer cells
Source: Breast Cancer Res. 2018 Sep 12;20:112. doi: 10.1186/s13058-018-1028-5 (PMC6134690; doi:10.1186/s13058-018-1028-5)
Supplement: Supplementary file 5 — Figure S5. NgBR knockdown attenuated EGF-stimulated signaling and ERα phosphorylation in T47D-TamR cells. (A) T47D-TamR cells were transfected with siNgBR and treated with EGF (100 ng/mL) for 5 min. Downstream signaling of the EGF pathway was determined using western blot assay. (B) Quantitative analysis of phosphorylated proteins presented in Additional file 10: Figure S6A were carried out using ImageJ and were normalized to total proteins. The data are from three separate repeated experiments and are presented as the mean ± SD (*p < 0.05, n = 3). (PDF 310 kb) [file 13058_2018_1028_MOESM5_ESM.pdf]

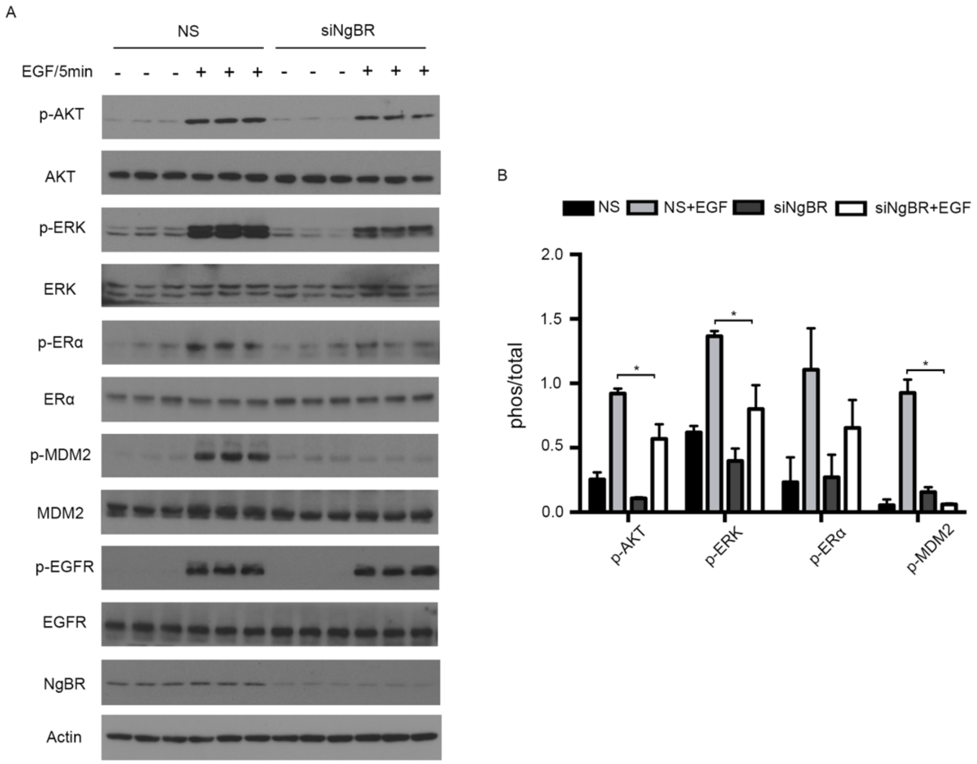

Figure S5 NgBR knockdown attenuated EGF-stimulated signaling and ERα phosphorylation in T47D-TamR cells. (A) T47D-TamR cells were transfected with siNgBR and treated with EGF (100ng/mL) for 5 minutes. Downstream signaling of EGF pathway was determined using western blot assay. (B) Quantitative analysis of phosphorylated proteins presented in Figure S6A were carried out using ImageJ and were normalized to total proteins. The data were repeated in three separate experiments, and are presented as the mean ± SD of. (* p<0.05, n=3).
