# Supplementary material for: Nogo-B receptor increases the resistance to tamoxifen in estrogen receptor-positive breast cancer cells
Source: Breast Cancer Res. 2018 Sep 12;20:112. doi: 10.1186/s13058-018-1028-5 (PMC6134690; doi:10.1186/s13058-018-1028-5)
Supplement: Supplementary file 10 — Figure S6. Relapse-free survival (RFS) in patients with ERα-positive breast cancer (n = 343). NgBR (NUS1) mRNA expression data were retrieved from the GSE6532 database. Kaplan–Meier analysis revealed significantly reduced RFS (p < 0.05) in patients with high NgBR expression in tumors (n = 189) as compared to patients with low NgBR expression in tumors (n = 154). (PDF 109 kb) [file 13058_2018_1028_MOESM10_ESM.pdf]

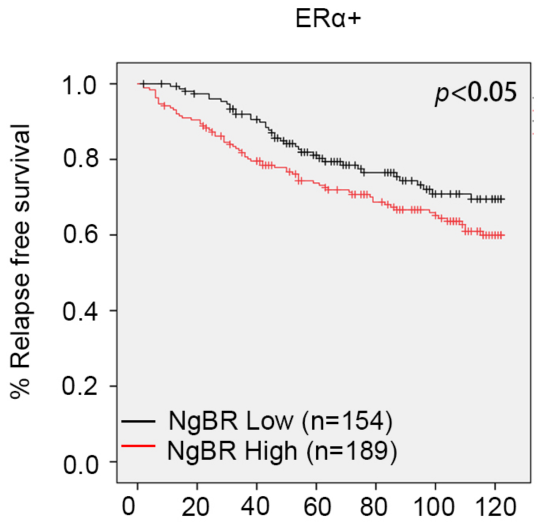

Figure S6 The relapse free survival of ERα positive breast cancer patients (n=343). NgBR (NUS1) mRNA expression data were retrieved from GSE6532 database. Kaplan-Meier analysis revealed a significantly reduced relapse free survival (RFS) ($p<0.05$) for patients with high NgBR expression in tumors (n=189) as compared to patients with low NgBR expression in tumors (n=154).
